# Supplementary material for: Interleukin 6 SNP rs1818879 Regulates Radiological and Inflammatory Activity in Multiple Sclerosis
Source: Genes (Basel). 2022 May 17;13(5):897. doi: 10.3390/genes13050897 (PMC9141517; doi:10.3390/genes13050897)
Supplement: Supplementary file 1 [file genes-13-00897-s001.zip › genes-1685663-supplementary.pdf]

Supplementary Materials:

Supplementary Table S1. Variance of the first six principal components in PCA.

	VE	PVE	CVE
1	6,673	24,715	24,715
2	5,588	20,696	45,410
3	2,625	9,723	55,134
4	1,593	5,902	61,035
5	1,378	5,103	66,138
6	1,141	4,226	70,364

Supplementary table Legend: principal component (PC); Variance Explained (VE); Percentual Variance Explained (PVE); Cumulative Variance Explained (CVE).

Supplementary Table S2. Logistic regression analysis between PCs and SNP rs1818879 group.

PC	*p* **value**	***β-coefficient***	*SE*
**PC1**	0.018*	0.27	0.11
**PC2**	0.70	0.03	0.075
**PC3**	0.89	-0.02	0.11
**PC4**	0.33	-0.14	0.15
**PC5**	0.62	-0.09	0.18
**PC6**	0.66	0.08	0.19

Supplementary table Legend: logistic regression to assess the association between rs1818879 and the first six PCs. (*) denotes statisticalsignificance ($p < 0.05$). Abbreviations: principal component (PC), standard error (SE).
